# Reference ranges for rotational thromboelastometry in male Sprague Dawley rats

**DOI:** 10.1186/s12959-017-0154-0

**Published:** 2017-12-28

**Authors:** Mariana Vigiola Cruz, Jenna N. Luker, Bonnie C. Carney, Kathleen E. Brummel-Ziedins, Maria-Cristina Bravo, Thomas Orfeo, Jason H. Chen, Lauren T. Moffatt, Jeffrey W. Shupp

**Affiliations:** 1 Firefighters’ Burn and Surgical Research Laboratory, MedStar Health Research Institute, Washington, DC USA; 2 The Burn Center, MedStar Washington Hospital Center, Washington, DC USA; 3 Department of Surgery, MedStar Georgetown University Hospital, Washington, DC USA; 4 Department of Biochemistry, University of Vermont College of Medicine, Colchester, VT USA; 5 Department of Surgery, MedStar Washington Hospital Center, 110 Irving Street, NW; Suite 3B-55, Washington, DC 20010 USA

**Keywords:** Coagulation, Sprague Dawley rat, Thromboelastometry, ROTEM, Reference range

## Abstract

**Background:**

A functional coagulation assay was used to investigate the extrinsic pathway of coagulation on citrated whole blood samples from healthy adult male Sprague Dawley rats using the mini cup and pin system.

**Methods:**

Reference values for coagulation parameters from forty-three animals were calculated using data obtained from the ROTEM® *delta* hemostasis analyzer with the EXTEM test.

**Results:**

The following ranges, presented as the 2.5–97.5 percentiles, were established: CT [18–77], CFT [20–80], α [73–86], MCF [53–70], and ML [1–22], along with others.

**Conclusions:**

These reference ranges can be used in future studies in rats to identify clinically significant coagulopathies.

## Background

Viscoelastic hemostatic assays are useful point of care tools in guiding therapy in trauma and cardiac surgery patients, as well as patients with other perioperative bleeding disorders or risk factors for coagulopathy [1, 2]. Sprague Dawley rats are commonly used in experimental models, including those examining the coagulopathy resultant from trauma and hemorrhagic shock. Thromboelastometry and thromboelastography have been applied in various experimental protocols for evaluation of clot formation and fibrinolysis in rats [3–6], and recognition of the utility of research involving these viscoelastic hemostatic assays has made their use more commonplace. Despite the widespread use of rodent models in coagulation studies, there is a lack of reference ranges for thromboelastometric parameters in the literature for Sprague Dawley rats. Lang et al. have described reference ranges in humans following a multi-centre approach employing blood donors, clinical personnel and other volunteers [7] and these ranges are used as the basis for treatment of coagulopathies in clinical care. Additionally, significant inter-species differences in the coagulation profiles between humans and various experimental animals have been previously reported [8, 9]. This established inter-species variation underscores the importance of defining reference intervals for individual species, and implies that reference ranges that define coagulopathies in one species do not do so in another. Additionally, often, when small animals are used, the use of the mini cup and pin system is required due to the obtainment of small volume aliquots at multiple time points. There are no established human reference ranges in the mini cup and pin system that are available for comparison, although it is established that differences exist between the two systems [10].

Clot formation in vivo is composed of two major pathways, extrinsic and intrinsic, which converge to regulate a complex biomolecular cascade leading to coagulation and fibrinolysis. The Extrinsic Tissue Factor pathway is tested by the EXTEM assay by ROTEM®. The results of this assay may be influenced by extrinsic coagulation factors, platelets and fibrinogen [7, 11]. The purpose of this study was to analyze citrated whole blood from a cohort of healthy rats to determine baseline thromboelastometry parameters as demonstrated by the EXTEM test in an effort to establish normal reference ranges that can be used to diagnose clinically significant coagulopathies in animal research.

## Methods

### Animals

The MedStar Health Research Institute Institutional Animal Care and Use Committee (IACUC) reviewed and approved all procedures and animal work described. Adult male Sprague Dawley rats weighing 300–350 g with pre-inserted tunneled jugular venous cannulas were obtained from Envigo (Dublin, VA and Indianapolis, IN, USA). Animals had free access to food and water and were quarantined for 7 days, housed singly, and maintained per facility standard operating procedures for cannulated animals prior to testing. There was a period of >12 days between the cannulation surgical procedure and the blood sampling collection described below, during which any anesthesia or analgesia from the cannulation procedure was no longer present.

### Blood sampling

A total of forty three male Sprague Dawley rats were analyzed in this study. Animals were anesthetized with 5% inhaled isoflurane gas in an induction chamber (EZ-anesthesia, Braintree, MA) for 5 min. In the absence of reflexes, animals were transferred to a nose cone on 2–3% isoflurane prior to sampling. Tunneled jugular venous cannulas were accessed using a 20G needle on a 1.0 mL Luer-lock syringe. Blood was drawn to waste approximately 100 μL, and the following 200 μL were collected for analysis and immediately aliquoted into 100 uL volumes in microtubes containing 11 μL of 3.8% sodium citrate (BD Life Sciences, Franklin Lakes, NJ USA). The cannulas were saline locked, secured with titanium clips and the animals were recovered from anesthesia. Samples were processed for thromboelastometry within five minutes of collection. All animals used in this study were part of a cohort of rats that would be used in a future study of coagulopathy after burn injury. The number of rats used in this experiment was not calculated based on a power calculation specific to the present reference range experiment, but was based on the amount of animals required for a future experiment.

### Rotem® system

**Fig. 1:**
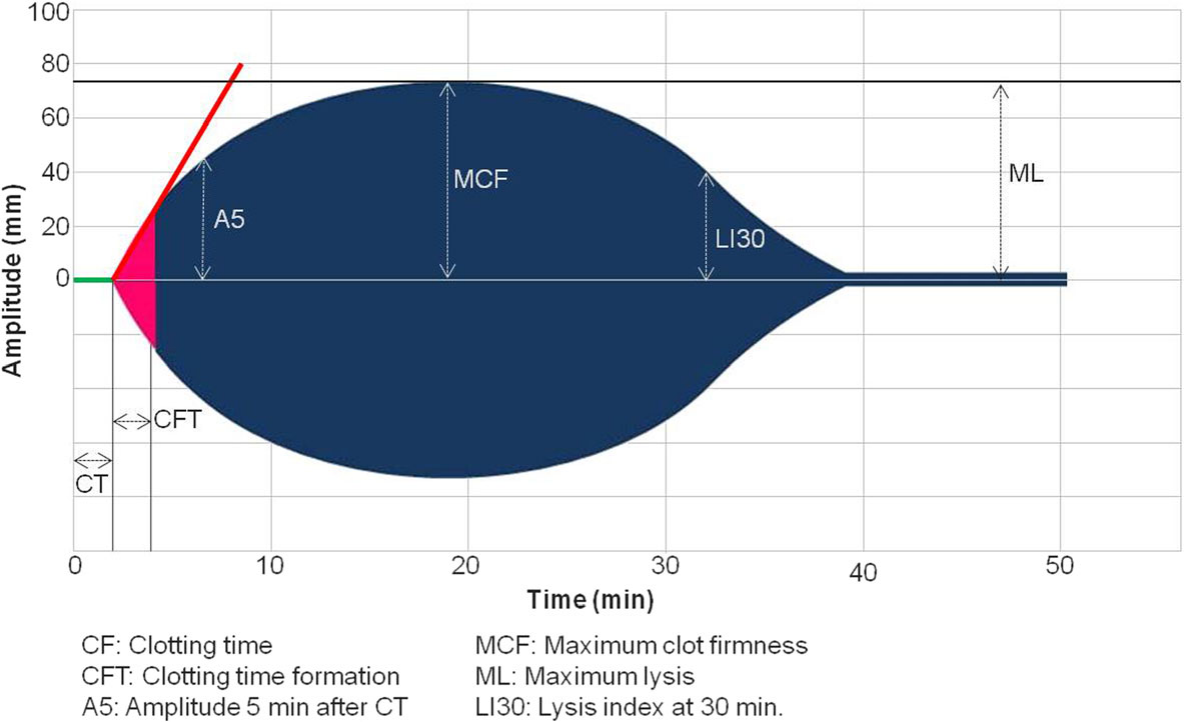
Representation of ROTEM® tracing (TEMogram) displaying key parameters

ROTEM® *delta* (Tem Systems Inc., Munich, Germany) is a rotational thromboelastometry hemostasis analyzer that provides quantitative and qualitative assessment of clot formation from whole blood samples by measuring kinetic changes of the clot elasticity. The ROTEM® system employs a cup and a vertical axis, which is supported by a high precision ball bearing and oscillates from left to right at an angle of 4.75°. A disposable plastic pin is attached to the axis and 105 μL of blood sample are added to a disposable plastic cup, which is then placed onto the measurement channel, immersing the pin within the sample. A mirrored plate at the upper end of the axis, a light source, and a chip sensor detects the rotation optically. As a clot forms and becomes firm, attaching the cup to the pin, oscillation is reduced. The ROTEM® system generates a reaction curve termed a “TEMogram” and calculates different parameters assessing clot kinetics and firmness [1, 7, 11] (Fig. 1).

### Extem assay

To analyze baseline coagulation function, rotational thromboelastometry was conducted in single determination according to the manufacturer’s protocol using the mini-cup and pin system with the EXTEM test (Tem Systems Inc., Munich Germany), which mildly activates coagulation via extrinsic tissue factor to standardize and accelerate in vitro clot formation. Whole blood collected in sodium citrate tubes was maintained at 37 °C. Following manufacturer’s recommendations, 7 μL of star-TEM reagent (0.2 M CaCl_2_) and 7 μL of EXTEM reagent (tissue factor) were manually pipetted into the mini-cups, followed by 105 μL of warm citrated whole blood. The blood and reagents were adequately mixed by manual pipetting to allow for activation of the extrinsic pathway. The cup holder was then placed onto the measuring position and the test was initiated. All ROTEM® assays were run for 120 min at 37 °C.

### Rotem® parameters

ROTEM® utilizes various parameters to describe the dynamics of clot formation and degradation, as well as size and firmness of the formed clot (Fig. 1).

The clotting time (CT, in seconds) describes the time from the start of measurement until the initiation of clot formation to an amplitude of 2 mm. The clot formation time (CFT, in seconds) describes the period from initiation of clotting until a clot firmness equivalent to a 20 mm amplitude is reached, relating to fibrin polymerization and clot stabilization. The alpha angle (α, in degrees), which also describes clot kinetics, is given by the angle between the horizontal axis and tangential line to the TEMogram curve through the 2 mm amplitude point. Clot amplitudes at 5–30 min (A5–A30, in millimeters) express the clot firmness at the respective time points after CT. Maximum clot firmness (MCF, in millimeters) is a measure of firmness and quality of the clot defined by the highest amplitude reached before the clot is dissolved, representing the maximal firmness that the clot achieves during the assay. The maximum clot elasticity (MCE, in dynes/centimeters [2]), calculated from the MCF, represents a value related to the physical clot elasticity and may assist in additional interpretation of MCF results. Maximum clot velocity (MAXV, in millimeters/min) is the maximum of the first derivative of the curve, and the Time to Maximum Velocity (MAXV-t, in seconds) is the period from test start until the MAXV is reached [1, 7, 10, 11].

Clot lysis can be measured by many ROTEM parameters. Maximum Lysis (ML, described in percent of MCF) demonstrates the maximum lysis detected during the analysis. The clot lysis indices at 30–60 min (LI30–LI60, in percent of MCF) express the residual clot firmness at the respective time points from CT, describing the progress of fibrinolysis. Additional parameters may be calculated by the ROTEM® software for further research applications [1, 7].

### Statistics

For each parameter, descriptive statistics were run to identify the mean, median, standard deviation, and 2.5- and 97.5 percentile confidence intervals to create a reference range. The use of the above statistics depends on normally distributed data, which was confirmed using D’Agostino and Pearson normality tests. Histograms were constructed to view the distribution of data. All statistic tests were run using Graphpad prism software.

## Results

**Fig. 2:**
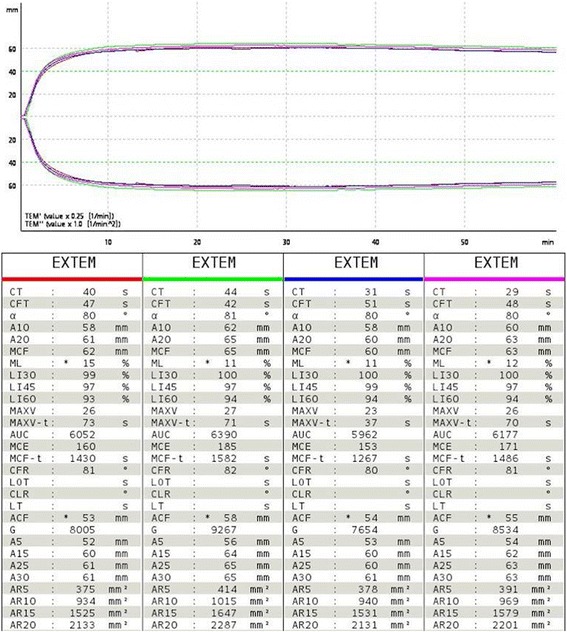
Sample TEMogram and ROTEM® report for four channels simultaneously run with whole blood samples from four different animals with EXTEM test

**Fig. 3:**
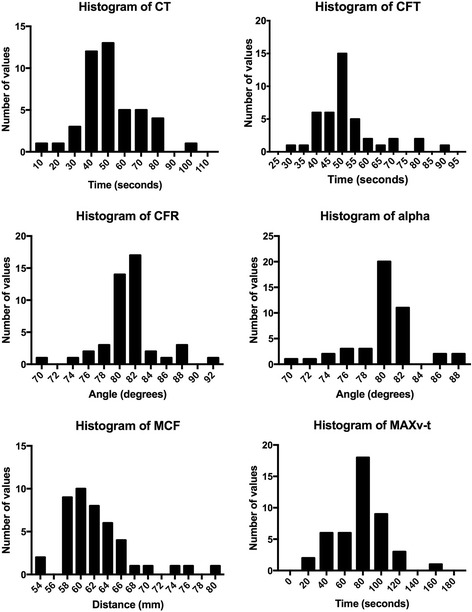
Representative histograms for ROTEM® parameters of coagulation and fibrinolysis with EXTEM test demonstrating normal distributions of data

Representative ROTEM® traces from individual animals are shown in Fig. 2. Descriptive statistics and histograms indicated mostly normal distribution of the ROTEM® results by D’Agostino and Pearson normality test (representative histograms are shown in Fig. 3). Mean and median values were calculated from individual animal values for each EXTEM parameter, and standard deviation was calculated to formulate reference ranges. Reference ranges are presented as the 2.5- and 97.5-percentiles in Table 1, as is standard for the reporting of human reference ranges [7].

**Table 1:** Calculated reference ranges presented as the 2.5- and 97.5-percentiles for commonly used ROTEM® parameters of coagulation and fibrinolysis with EXTEM test

Parameter	Median	Mean	Reference range
CT (s)	47	48	18–77
CFT (s)	50	50	20–80
CFR (°)	81	81	73–88
α (deg)	80	80	73–86
MCF (mm)	61	62	53–70
MCF-t (s)	1491	1540	894–2186
MCE (dynes/cm^2^)	153	158	86–230
MAXV (mm/min)	24	24	14–34
MAXV-t (s)	81	76	28–123
ML (%)	12	12	1–22
LI30 (%)	100	100	98–102
LI45 (%)	98	96	93–102
LI60 (%)	94	94	87–101
AUC (mm^2^)	6023	6081	5277–6885
ACF (mm)	54	54	45–62
A5 (mm)	51	51	38–64
A10 (mm)	57	57	47–66
A15 (mm)	59	59	50–68
A20 (mm)	60	60	52–69
A25 (mm)	60	61	52–69
A30 (mm)	61	61	53–69
AR5 (mm^2^)	369	363	244–482
AR10 (mm^2^)	913	910	675–1145
AR15 (mm^2^)	1486	1483	1186–1780
AR20 (mm^2^)	2076	2080	1702–2458

## Discussion

Numerous studies have analyzed the clinical use of ROTEM and thromboelastography (TEG) in a variety of settings, including trauma, cardiac surgery and liver transplantation. The clinical applications of these viscoelastic assays in humans are well established for understanding individual coagulopathic and fibrinolytic profiles in patients, but their use in translational research related to hemostasis involving animal models is challenged by a scarceness of data indicating reference ranges. The importance of understanding clotting parameters in animals has been highlighted in recent publications exploring larger animal models for hemostatic alterations in cardiovascular surgery and haemorrhage [12, 13]. Rats can be useful models for hemostatic analysis in diverse pathologic states, including those commonly managed clinically with the aid of TEG or ROTEM.

No prior reference ranges for rotational thromboelastometry have been reported in Sprague Dawley rats. Considering the importance of accurate and translatable animal models for the study of coagulation and fibrinolysis physiology, particularly in investigating pathologic states, the authors wished to address this paucity in the literature to provide a guide for interpretation of rotational thromboelastometry data. The selection of this system and the EXTEM test, both widely established in the experimental and clinical settings, may allow for broader applicability of the results obtained from this study. As described by Siller-Matula et al., EXTEM results are more similar among species than NATEM or INTEM data, implying that the EXTEM test may be the ROTEM assay that may provide the most translational data [8].

Each parameter measured by ROTEM can assist in determining the need for transfusion and the choice of resuscitative fluid or therapeutic intervention. In humans, well-established reference ranges can indicate abnormalities in clot formation, strength, or breakdown. A prolonged clotting time parameter suggests the need to substitute clotting factors, such as fresh frozen plasma, factor concentrates, or anticoagulant inhibitors. A prolonged clot formation time suggests the need for fibrinogen or thrombocyte concentration, which may be administered as cryoprecipitate, fibrinogen concentrate, or fresh frozen plasma. Decreased maximum clot firmness, or low MCF, suggests potential bleeding, and may indicate the need for administration of fibrinogen, thrombocyte concentrate, and/or antifibrinolytics. Alternatively, a reduced alpha angle, shortened CFT, and high MCF indicate hypercoagulation. The lysis index parameters assist in decision making regarding the use of anti-fibrinolytic agents [1, 11, 14].

In previous literature, the differences between the mini-cup versus the conventional cup systems for ROTEM® have been reported as considerable, with mini-cup results demonstrating lower amplitudes and higher variability in the parameters reflecting the kinetics of clot formation in human blood [15]. Although the mini-cup model may be applicable beyond small rodent models in the pediatric patient population, reference ranges do not exist for this system in humans either. Despite the paucity of data from which to compare our work; the reference ranges that we provide are in agreement with the values reported by Griffin et al. They report, in a group of Sprague Dawley rats under isoflurane anesthesia (*n* = 6), a mean value of CT = 38, CFT = 38, α = 83, CFR = 84, maxV = 39, and ML = 8. All of these values are within the normal ranges that we have defined. Darlington et al. also report “baseline” ROTEM parameters for a group of Sprague Dawley rats (*n* = 7–11) that agree with our findings (MCF = 55, CT = 48, α = 73) [16].

A possible limitation to consider in this study is the potential for differences in results stemming from variability in the processing of samples and rotational thromboelastometry assays. In a prior multi-center comparative analysis of human blood tested for coagulation parameters, no significant differences in results were reported despite existing disparities in the pre-analytical factors, such as sampling technique or variations in the concentration of anticoagulant in aliquots [7]. Nonetheless, precautions to minimize machine or operator variability were followed. The device utilized was kept per manufacturer standards with weekly maintenance and calibration tests, as well as periodic professional servicing. EXTEM reagents were stored per manufacturer guidelines and utilized prior to their expiration date. The performance of collection and processing of samples was limited to two individuals. Another limitation in this study is the number of animals that was used in this experiment. Because the number of animals was defined by a power calculation for a different experiment for which the animals were used, 43 animals was not a specific number required for this study. Despite this limitation, the reference ranges that are presented here are much more robust than others that have been reported in cats (*n* = 25) [9], sheep (*n* = 6), pig (*n* = 6), and rabbit (*n* = 6) [8].

## Conclusions

Following rotational thromboelastometry assays with mini-cup and pin system and EXTEM test in a cohort of male Sprague Dawley rats, we have formulated a standard and reproducible protocol and a series of species-specific reference ranges for commonly used parameters to assess coagulation and fibrinolysis in a rat animal model. The established reference indices may serve as guidelines in experimental design and analysis for future diagnostic and translational thromboelastometry assays in rodents.

## Abbreviations

A10: firmness at 10 min
A15: firmness at 15 min
A20: firmness at 20 min
A25: firmness at 25 min
A30: firmness at 30 min
A5: firmness at 5 min
ACF: actual clot firmness
AR10: area under the curve from CT to 10 min
AR30: area under the curve from CT to 30 min
AR5: area under the curve from CT to 5 min
AUC: area under first derivative curve
CFR: clot formation rate
CFT: clot formation time
CT: clotting time
IACUC: Institutional Animal Care and Use Committee
LI30: maximum lysis detected at 30 min
LI45: maximum lysis detected at 45 min
LI60: maximum lysis detected at 60 min
MAXV: maximum clot velocity
MAXV-t: time to maximum velocity
MCE: maximum clot elasticity
MCF: maximum clot firmness
MCF-t: maximum clot firmness-time
ML: maximum lysis
ROTEM®: Rotational Thromboelastometry
α: alpha angle

## References

[1] Görlinger K, Dirkmann D, Hanke AA, Gonzalez E, Moore HB, Moore EE (2016). Rotational Thromboelastometry (ROTEM®), in Trauma Induced Coagulopathy.

[2] Letson HL, Dobson GP (2015). Correction of acute traumatic coagulopathy with small-volume 7.5% NaCl adenosine, lidocaine, and Mg2+ occurs within 5 minutes: a ROTEM analysis. J Trauma Acute Care Surg.

[3] Park KH, Kee KH, Kim H (2013). Effect of hypothermia on coagulatory function and survival in Sprague–Dawley rats exposed to uncontrolled haemorrhagic shock. Injury.

[4] Torres LN, Sondeen JL, Ji L (2013). Evaluation of resuscitation fluids on endothelial glycocalyx, venular blood flow, and coagulation function after hemorrhagic shock in rats. J Trauma Acute Care Surg.

[5] Rezende-Neto JB, Lage-Alves R, Carvalho Jr M, et al. Vagus nerve stimulation improves coagulopathy in hemorrhagic shock: a thromboelastometric animal model study. J Trauma Manag Outcomes. 2014;8(15) 10.1186/1752-2897-8-15 PMC4169132 25243020

[6] Shoffstall AJ, Atkins KT, Groynom RE (2012). Intravenous hemostatic nanoparticles increase survival following blunt trauma injury. Biomacromolecules.

[7] Lang T, Bauters A, Braun SL (2005). Multi-centre investigation on reference ranges for ROTEM thromboelastometry. Blood Coagul Fibrinolysis.

[8] Siller-Matula JM, Plasenzotti R, Spiel A (2008). Interspecies differences in coagulation profile. Thromb Haemost.

[9] Marly-Voquer C, Riond B, Jud Schefer R (2017). Reference values for rotational thromboelastometry (ROTEM) in clinically healthy cats. J Vet Emerg Crit Care.

[10] Griffin MJ, Letson HL, Dobson GP. Buprenorphine analgesia leads to coagulopathy and increased plasma fibrinogen in healthy rats: implications for small animal research. Shock. 2016; Epub ahead of print 10.1097/SHK.0000000000000821 27984521

[11] Tem. In: GmbH TI, editor. ROTEM(R) delta Operating Manual. Munich: Tem Innovations; 2016.

[12] Mizuno T, Tsukiya T, Takewa Y, et al. Differences in clotting parameters between species for preclinical large animal studies of cardiovascular devices. J Artif Organs. 2017; 10.1007/s10047-017-1003-4 29124459

[13] Schott U, Kander T, Bentzer P. Effects of Dextran-70 and albumin on coagulation in experimental hemorrhage in the Guinea pig. Shock. 2017; 10.1097/SHK.0000000000001025 PMC6072367 29049137

[14] Walsh M, Fritz S, Hake D (2016). Targeted Thromboelastographic (TEG) blood component and pharmacologic hemostatic therapy in traumatic and acquired coagulopathy. Curr Drug Targets.

[15] Haas T, Spielmann N, Dillier C (2015). Comparison of conventional ROTEM® cups and pins to the ROTEM® cup and pin mini measuring cells (MiniCup). Scand J Clin Lab Ivest.

[16] Darlington DN, Craig T, Gonzales MD (2013). Acute coagulopathy of trauma in the rat. Shock.

